# Comparative analysis of lumbar quadratus lumborum block and epidural block for analgesia in uterine surgery at Dr. Soetomo Hospital, Surabaya

**DOI:** 10.25122/jml-2023-0196

**Published:** 2023-11

**Authors:** Usamah Usamah, Christrijogo Sumartono, Mariza Fitriati, Belindo Wirabuana, Brahmana Askandar Tjokroprawiro

**Affiliations:** 1 Department of Anesthesiology and Intensive Therapy, Faculty of Medicine, Universitas Airlangga, Dr. Soetomo Regional General Hospital, Surabaya, Indonesia; 2 Faculty of Medicine, Universitas Airlangga, Dr. Soetomo Regional General Hospital, Surabaya, Indonesia

**Keywords:** epidural block, NRS, opioids, postoperative pain, quadratus lumborum block

## Abstract

Over 80% of surgical patients experience postoperative pain, which, if inadequately managed, can lead to complications, prolonged rehabilitation, chronic pain, and decreased quality of life. Epidural block and quadratus lumborum block are techniques commonly used for postoperative pain management. This comparative analytic study aimed to analyze the differences in the analgesic effects of quadratus lumborum block and epidural block in uterine surgery at Dr. Soetomo General Hospital. The outcomes assessed were the numerical rating score (NRS) as a pain score and the administration of opioids as an adjuvant analgesic. Statistical analysis employed the Mann-Whitney test and Chi-square test. The study included 32 patients who underwent uterine surgery at Dr. Soetomo General Hospital and met the inclusion and exclusion criteria. Among the patients, 90.6% experienced mild pain, and 9.4% experienced moderate pain. Epidural blocks were performed in 50% of the patients, while quadratus lumborum blocks were performed in the other 50%. Additionally, 9.4% of the patients received opioids as adjuvant analgesics. The Mann-Whitney test revealed no significant difference in NRS between the epidural block and quadratus lumborum block groups (p-value>0.05). However, the Chi-square test indicated a significant difference in NRS between patients who received additional opioids as adjuvant analgesics and those who did not (p-value<0.00). There was no significant difference in NRS between patients who underwent epidural block and quadratus lumborum block as analgesic techniques.

## INTRODUCTION

Surgery often results in tissue damage and inflammation, leading to the experience of pain. The International Association for the Study of Pain (IASP) defines pain as an unpleasant sensory and emotional sensation caused by actual or potential tissue damage. Pain management is considered a fundamental human right by the World Health Organization and IASP [1, 2]. However, a report by the US National Institutes of Health in 2011 revealed that more than 80% of surgical patients experience postoperative pain, with less than 50% receiving adequate pain relief. Additionally, persistent postoperative pain affects approximately 2-10% of adult surgical patients, equating to millions of individuals worldwide [3]. Inadequate management of postoperative pain can lead to complications, prolonged rehabilitation, chronic pain, and a diminished quality of life. Conversely, effective pain management has been associated with shorter hospital stays, reduced healthcare costs, and improved patient satisfaction [4, 5].

Epidural block has long been a commonly utilized technique for postoperative pain management. Previous research has highlighted its benefits, including reduced cardiovascular, pulmonary, and gastrointestinal morbidity and lower mortality rates. For many years, epidural block has been considered the gold standard for postoperative pain management. However, recent studies have presented conflicting results, with some indicating a decrease in morbidity and others suggesting an increase. Furthermore, the risk of neurological complications associated with epidural block is higher than previously believed, encompassing neuroaxial hematoma, epidural abscess, epidural hematoma, and even mortality [3].

The quadratus lumborum block, a modified version of the transversus abdominis plane (TAP) block commonly employed in abdominal surgery, is a relatively novel technique. Previous studies have demonstrated its ability to provide a wide range of blocks spanning from T7-L1, effectively reducing postoperative pain, minimizing opioid usage, and promoting expedited patient recovery. Research conducted by She *et al*. [6] indicated that the quadratus lumborum block yields a less intense block compared to the epidural block but with a longer duration and fewer side effects [6].

Pain, as defined by the IASP, is an unpleasant sensory and emotional experience caused by actual or potential tissue damage. It is a subjective experience influenced by biological, psychological, and social factors [2].

Pain signal delivery involves transduction, transmission, modulation, and perception. Transduction occurs when pain-causing substances are released due to tissue or organ damage, stimulating nerve endings. The subsequent transmission involves delivering the stimulus or signal along nerve fibers to the dorsal horn of the spinal cord. Modulation refers to signal amplification or suppression before the pain signal is processed and perceived in the cerebral cortex [7, 8].

During surgery, tissue trauma leads to the release of local proinflammatory mediators. Prostaglandins, leukotrienes, bradykinin, histamine, and 5-hydroxytryptamine (5HT) are released, sensitizing peripheral and central pain receptors and causing pain [4, 5]. In addition to pain, tissue damage triggers sympathetic response and neuroendocrine activation, resulting in symptoms such as tachycardia, hypertension, hyperglycemia, immunosuppression, platelet aggregation, and decreased regional blood flow or venous stasis [9].

Pain assessment can be conducted using the Numerical Rating Scale (NRS), which ranges from 0 to 10 or 0 to 100 to indicate pain severity, with 0 representing no pain and 10 or 100 representing the worst pain. Generally, a score of 4 is considered the threshold for determining whether a patient's pain is adequately managed or if further treatment is required [10].

Several factors can influence postoperative pain, including preoperative pain, anxiety, obesity, fear of surgery, and the type and duration of the surgical procedure. Previous studies have shown associations between surgical type, age, psychological distress, and postoperative analgesic medication consumption. Preoperative anxiety has been linked to higher-intensity postoperative pain, while depression and sleep disturbances have also been associated with intractable postoperative pain [9, 11, 12].

Regional anesthesia is a technique used to reduce sensation in a specific part of the body. It allows for targeted anesthesia during surgery or other invasive procedures while the patient remains conscious and able to breathe independently [13].

General anesthesia carries higher risks in certain patients due to hemodynamic fluctuations, airway and respiratory disorders, stress response, and potential complications, particularly in the respiratory system. Regional anesthesia is an alternative for these patients, as it does not require mechanical ventilation. Combining general anesthesia with regional anesthesia has been shown to reduce mortality, improve pain control, and lower the incidence of complications compared to general anesthesia alone. Additionally, regional anesthesia allows for more efficient transfer of patients to the post-anesthesia care unit, recovery room, or ward, as they can breathe independently, leading to faster postoperative recovery and discharge [13-15].

Regional anesthesia can be accompanied by sedation to enhance patient comfort and minimize trauma by preventing the patient from remembering the surgical procedure. In cases where regional anesthesia alone does not provide sufficient anesthesia for the surgical area, it can be converted to general anesthesia [13].

Regional anesthesia is believed to reduce sensitization by inhibiting the transmission of afferent nociceptive signals from the surgical area to the spinal cord or brain. Local anesthetic agents administered for nerve blocks can reduce acute inflammation, early cytokine production, and sensitization, alleviating surgical pain [16, 17]. Local anesthetics also act on autonomic nerves, causing vasodilation of peripheral blood vessels through sympathetic blockage in the posterior hypothalamus [18]. When combined with enhanced recovery after surgery protocols, regional anesthesia can decrease the need for opioids, thereby reducing opioid-related side effects such as sedation, nausea, vomiting, urinary retention, respiratory depression, bowel dysfunction, ileus, and sleep disturbances [19].

The quadratus lumborum block, a fascial plane technique, involves injecting a local anesthetic near the quadratus lumborum muscle to anesthetize the thoracolumbar nerves [20]. This regional anesthesia technique can be employed in various age groups, including pediatrics, adults, and pregnant women undergoing abdominal surgery [21].

Previous studies have demonstrated positive outcomes associated with the quadratus lumborum block. It has been shown to reduce opioid consumption by 48 hours after cesarean section and has an opioid-sparing effect when compared to the TAP block. Additionally, the use of the quadratus lumborum block has led to a decrease in the requirement for opioid resuscitation in lower abdominal surgery [20-22].

The epidural block is a regional anesthesia technique that involves the injection of a local anesthetic agent into the space between the dura mater and the spinal canal wall. This continuous cavity allows for analgesia to be limited to a specific dermatome (caudal anesthesia) or extended to a higher level (sacrolumbar epidural anesthesia), depending on the volume of the anesthetic agent injected into the sacral canal [23].

Epidural anesthesia is widely utilized in cesarean section surgeries due to its various advantages. It reduces the adverse effects of analgesic drugs, inhalation, or intravenous anesthesia on both the mother and baby. It also provides better control over the level of anesthesia compared to spinal block and reduces the incidence of hypotension and post-dural puncture headache [24]. Additionally, epidural block offers the flexibility of administering the drug as a one-time injection or continuously through an infusion pump, ensuring a longer-lasting analgesic effect. Other benefits include decreased cortisol levels, faster recovery of digestive function, reduced incidence of pulmonary embolism or deep vein thrombosis in the postoperative period, and shorter hospitalization time [25].

Despite these developments, there remains a scarcity of studies comparing the analgesic effects of epidural blocks *versus* quadratus lumborum blocks. Therefore, the objective of this study was to investigate and compare the analgesic effects of quadratus lumborum block and epidural block in uterine surgery.

### Research problem

Inadequate management of postoperative pain in surgical patients can lead to various complications, extended rehabilitation periods, chronic pain, and reduced quality of life. While epidural block and quadratus lumborum block are commonly employed techniques for postoperative pain management, there remains a need to comprehensively evaluate their comparative analgesic efficacy in the context of uterine surgery. This study aimed to address this gap by analyzing and contrasting the analgesic effects of epidural block and quadratus lumborum block in uterine surgery patients at Dr. Soetomo General Hospital. The investigation specifically focuses on the differences in NRS as a pain assessment tool and the utilization of opioids as adjuvant analgesics between the two techniques. The outcomes of this research have the potential to provide valuable insights into optimizing pain management strategies for uterine surgery patients, ultimately enhancing postoperative recovery and overall patient well-being.

## MATERIAL AND METHODS

### Design

This study employed a comparative analytic research design to analyze the difference in analgesic effects between quadratus lumborum block and epidural block in uterine surgery at Dr. Soetomo Hospital Surabaya. The surgeries involved either general or regional anesthesia. The anesthetic agents used were ropivacaine or bupivacaine. The required dose was 0.2-0.4 ml/kg of 0.2-0.5% ropivacaine or 0.1-0.25% bupivacaine on each side. The regional anesthesia techniques, namely the quadratus lumborum block and the epidural block, involved the application of local anesthetics either in the epidural space or around the quadratus lumborum muscle. The objective was to achieve a specific level of blockage, providing both anesthetic and analgesic effects.

### Population and sampling

The study population comprised patients who underwent uterine surgery at the Hospital Dr. Soetomo Surabaya in 2023, using either general or regional anesthesia. The sample consisted of patients who underwent uterine surgery at the Hospital Dr. Soetomo Surabaya in 2023 and met the inclusion criteria, expressed their willingness to participate as research subjects, and did not meet the exclusion criteria.

The inclusion criteria encompassed patients undergoing uterine surgery using general or regional anesthesia, receiving postoperative analgesic therapy with either epidural block or quadratus lumborum block, and postoperative analgesic therapy with Paracetamol drip at a dosage of 20 mg/kg BW every 8 hours. Additionally, research subjects were required to willingly participate in the study and possess the ability to understand and read Indonesian.

Patients were excluded if they had an infection in the needle insertion area for quadratus lumborum block or epidural block, bacteremia, allergies to local anesthetic agents, spinal anatomical abnormalities, unstable hemodynamics, blood clotting disorders, were undergoing anticoagulant therapy, had anxiety, depression, preoperative pain, sleep disorders, or were classified as grade 3 or higher in obesity. Patients allergic to Paracetamol were also excluded.

The sample size was calculated using J. Charan and T. Biswas formula [26], resulting in 16 subjects for each group, rounded up from the estimated size of 15.68. The consecutive sampling technique was employed, where all eligible subjects within the specified time frame were selected until the minimum sample size was achieved.

### Instruments

The independent variable in this study was the administration of either quadratus lumborum block or epidural block. The dependent variables measured were the NRS for pain assessment and opioid administration. The research was conducted at the Hospital Dr. Soetomo Surabaya from March to May 2023.

### Data collection

The research procedure involved preparing a research proposal and obtaining ratification from the research supervisor. Ethical clearance was then sought at the Hospital Dr. Soetomo Surabaya. Following ethical approval, data collection was carried out through history taking and completion of data collection sheets. Subsequently, the research data was processed and presented.

### Analysis

To process the collected data, we utilized IBM SPSS Statistics 26 software. The data underwent processing to ensure accuracy and consistency. Subsequently, the processed data were subjected to statistical analysis using appropriate tests based on the nature of the data. For nominal scale data, we employed the Chi-square comparison test. For interval scale data with a normal distribution, we performed the unpaired t-test. In cases where the interval scale data exhibited an abnormal distribution, we used the Mann-Whitney test. It is important to note that a significance level of p<0.05 was considered statistically significant, indicating a noteworthy result.

### Conceptual Framework

**Figure 1:**
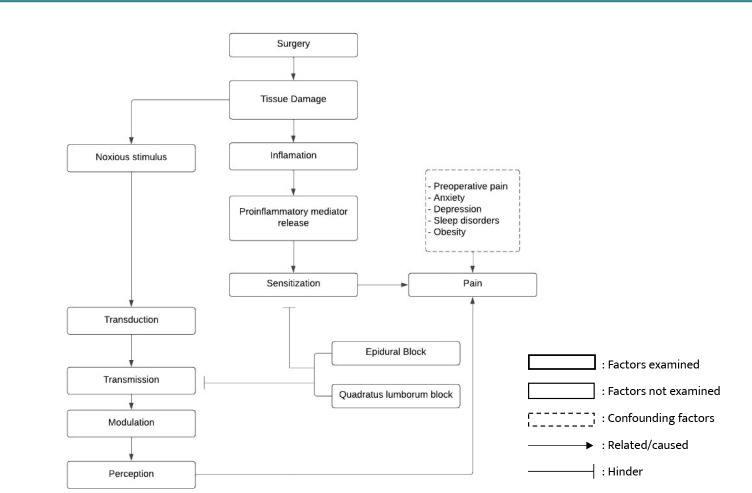
Conceptual framework

During surgery, tissue damage occurs, leading to inflammation. This inflammatory response releases proinflammatory mediators that cause both peripheral and central sensitization, resulting in the experience of pain. Simultaneously, the tissue damage acts as a stimulus, transmitted through afferent nerve endings to the dorsal horn of the spinal cord. Within the spinal cord, a process called modulation amplifies the signal. Ultimately, the impulse reaches the cerebral cortex, where it is perceived as pain (Figure 1).

Regional anesthesia techniques, such as the quadratus lumborum block and epidural block, involve the administration of local anesthetic agents into the epidural cavity or around the quadratus lumborum muscle. The primary objective is to achieve a targeted level of block, providing both anesthetic and analgesic effects. The local anesthetic agents work by inhibiting the process of transmission and sensitization, effectively reducing pain.

The application of quadratus lumborum block and epidural block in uterine surgery at Dr. Soetomo Hospital, Surabaya, aims to provide analgesic effects by interrupting the pain pathway. By administering local anesthetic agents to the epidural cavity or quadratus lumborum muscle, the transmission and sensitization processes are inhibited, resulting in reduced pain perception.

Several factors, such as preoperative pain, anxiety, depression, sleep disorders, and obesity, can influence postoperative pain and act as confounding variables in this study. Understanding the impact of these factors will help evaluate the effectiveness of the quadratus lumborum block and epidural block techniques in providing analgesic effects during uterine surgery.

## RESULTS

### Participants’ characteristics

Table 1 presents the characteristics of participants. A total of 32 patients were included in this study, with an average age of 43.6 years and an age range between 21 and 61. The mean BMI was 27.2±3.66. The NRS scores of the patients ranged from 1 to 4, with an average score of 1.6±0.9.

**Table 1:** Participants characteristics

Parameter	N (Min – Max) Mean ± SD
**Age**	32 (21–61) 43.6±9.8
**BMI**	32 (14.1–28.0) 27.2±3.66
**NRS Score**	32 (1–4) 1.6±0.9

Out of 32 patients, 16 (50%) received the epidural block analgesic method during uterine surgery, while an equal number of patients, 16 (50%), were provided with the quadratus lumborum block analgesic method (Table 2). This information sheds light on the distribution and utilization of analgesic methods for pain management in uterine surgery patients.

**Table 2:** Analgesic methods for uterine surgery

Analgesic	Quantity	Percentage (%)
Epidural Block	16	50
Quadratus Lumborum Block	16	50
**Total**	**32**	**100**

After analgesic administration, NRS levels were measured (Table 3). 29 patients (90.6%) experienced mild pain (NRS scores 1-3), while 3 patients (9.4%) reported moderate pain (NRS scores 4-7). This data reflects the distribution of pain levels among the patients following the analgesic intervention.

**Table 3:** NRS levels

NRS Level	Quantity	Percentage (%)
Mild Pain	29	90.6
Moderate Pain	3	9.4
**Total**	**32**	**100**

According to Table 4, 3 patients (9.4%) received opioid analgesic methods, while 29 patients (90.6%) did not receive opioid analgesic methods after their NRS scores were measured following uterine surgery. This information highlights the utilization of opioid analgesics in managing postoperative pain for uterine surgery patients at Dr. Soetomo General Hospital.

**Table 4:** Opioid analgesic methods

Opioid	Quantity	Percentage (%)
No	29	90.6
Yes	3	9.4
**Total**	**32**	**100**

### Data analysis

The data presented in Table 5 indicates a P-value less than the alpha threshold of 0.05 (5%). This suggests that the data did not follow a normal distribution, making it suitable for non-parametric testing, specifically the Mann-Whitney test.

**Table 5:** Kolmogorov-Smirnov test results (data normality)

Variable	p-value	
	Epidural	QL Block
NRS	<0.005	<0.005

### Comparison/difference test

In this study, two comparison tests were conducted: the Mann-Whitney test for interval-scaled data with non-normal distribution and the Chi-square test for nominal-scaled data.

### Mann-Whitney test analysis

The test for differences between the effects of epidural analgesia block and quadratus lumborum block was performed using the Mann-Whitney test. The Mann-Whitney test aims to determine whether there is a significant difference in the means of two independent samples. It is an alternative to the independent t-test when the research data does not follow a normal distribution.

The Mann-Whitney test (Table 6) indicated a p-value of 0.118, exceeding the 0.05 significance threshold. This result supports the null hypothesis, suggesting no significant difference in the analgesic efficacy of epidural *versus* quadratus lumborum blocks.

**Table 6:** Mann-Whitney test results

	NRS
Mann-Whitney U	91.5
Wilcoxon W	227.5
Z	-1.563
Asymp. Sig. (2 tailed)	0.118

### Chi-Square test analysis

The Chi-square test was used to examine comparative hypotheses between two samples when the data was on a nominal scale. In this study, the Chi-square test was conducted to investigate the difference in opioid administration requirements between patients who received epidural analgesia block and those who received quadratus lumborum block. This analysis also considered the additional opioid analgesic method.

Analysis of opioid administration requirements (Table 7) revealed no significant difference between epidural and quadratus lumborum blocks in uterine surgery patients at Dr. Soetomo Regional General Hospital (p=0.069).

**Table 7:** Chi-square test results

Analgesic	Opioid						p-value
	No		Yes		Total		
	f	%	f	%	f	%	
Epidural Block	16	55.2	0	0	16	50	0.069
Quadratus Lumborum Block	13	44.8	3	100	16	50	
**Total**	**29**	**100**	**3**	**100**	**32**	**100**	

## DISCUSSION

This study assessed the analgesic efficacy of epidural block (EB) and quadratus lumborum block (QLB) in 32 patients undergoing uterine surgery at Dr. Soetomo Surabaya Hospital. The study cohort comprised 32 patients with a mean age of 43 years and a mean BMI of 22.72. The NRS scores indicated that most patients experienced mild postoperative pain, which could be attributed to effective pain management strategies.

Postoperative pain is a complex phenomenon involving somatic and visceral pain pathways. Somatic pain is localized and arises from myelin-rich fibers, while visceral pain is diffuse and involves unmyelinated and thinly myelinated fibers that converge before reaching the spinal cord. Therapeutic interventions for pain management target these afferent pathways to alleviate pain [27, 28]. Inadequate pain control not only affects patients' physical well-being but also leads to various negative consequences, including impaired daily activities, sleep disturbances, mood changes, and an increased risk of chronic pain [29].

Opioid analgesics have traditionally been a cornerstone of acute postoperative pain management. However, their use comes with the risk of side effects and the potential for addiction and substance abuse. The study results indicate a significant correlation between patients' NRS scores and opioid administration, highlighting the effectiveness of opioids in managing post-surgical pain. Nevertheless, due to the rising concerns surrounding opioid misuse, there is a growing need for alternative pain management strategies, such as multimodal approaches [29].

Epidural block and quadratus lumborum block were compared in this study as regional analgesic techniques. The results indicated no significant difference in analgesic effects between the two methods. Both techniques have their advantages and considerations. Epidural block is established for laparotomy analgesia, while QLB is a newer approach derived from TAP block and has gained popularity for abdominal surgery due to its potential to reduce postoperative pain and opioid consumption [20, 21, 30].

Previous research has demonstrated that QLB offers longer-lasting analgesia and reduced opioid demand compared to other methods [31-33]. However, some studies have reported contrasting results, showing differences in pain scores and drug requirements between the QLB and EB groups [6, 34]. These discrepancies could be attributed to variations in study design, patient characteristics, and other factors.

### Analysis of research results and limitations

The NRS variable was analyzed using the Mann-Whitney comparison test in both the epidural block and quadratus lumborum block groups. The results revealed no statistically significant difference between the two groups, indicating that both methods had comparable analgesic effects in patients undergoing uterine surgery. Furthermore, the Chi-square test yielded a p-value of 0.069, which suggests that there was no significant disparity in the requirement for opioid administration between patients who received epidural block and quadratus lumborum block as analgesic techniques in uterine surgery at Dr. Soetomo Hospital Surabaya.

In the implementation of this research, there were several shortcomings due to research limitations, including:

1. The study was only conducted in one health facility, so it may be different if used in other health facilities.
2. There was no measuring instrument to ensure the quadratus lumborum block works.
3. Each sample has a different pain threshold.

## CONCLUSION

The findings in this study lead to several important conclusions regarding pain management in uterine surgery.

1. There was a significant relationship between the NRS scores of uterine surgery patients who received quadratus lumborum block.
2. There was a significant relationship between the NRS scores of uterine surgery patients who received epidural blocks.
3. There was no significant difference in NRS scores between uterine surgery patients using epidural block and those using quadratus lumborum block.
4. There was no significant difference in opioid requirement between uterine surgery patients using epidural block and those using quadratus lumborum block.

For future studies with similar objectives, it is recommended that researchers allocate more time and expand the data coverage to include a larger sample size. Additionally, it is advised to consider confounding variables when analyzing the correlation between them. Furthermore, it is hoped that this study can serve as a valuable reference and source of information for other researchers.
